# Recombinant human alpha-N-acetylglucosamine-6-sulfatase delivered to Sanfilippo D mice with repeated intracerebroventricular injections corrects CNS pathology

**DOI:** 10.1371/journal.pone.0328941

**Published:** 2025-07-25

**Authors:** Grant L. Austin, Feng Wang, Steven Q. Le, Alexander Sorensen, Shan Li, Lai C. Foong, Srikanth Singamsetty, Jill Wood, Tsui-Fen Chou, Patricia I. Dickson

**Affiliations:** 1 Department of Pediatrics, Washington University School of Medicine, St. Louis, Missouri, United States of America; 2 Division of Biology and Biological Engineering, California Institute of Technology, Pasadena, California, United States of America; 3 Phoenix Nest Inc., Brooklyn, New York, United States of America; 4 Proteome Exploration Laboratory, Beckman Institute, California Institute of Technology, Pasadena, California, United States of America; University of Utah Medical Center: University of Utah Hospital, UNITED STATES OF AMERICA

## Abstract

Mucopolysaccharidosis type IIID (MPS IIID; Sanfilippo D) is caused by biallelic pathogenic variants in N-acetylglucosamine-6-sulfatase (GNS), which participates in catabolism of heparan sulfate (HS) glycosaminoglycans. Characterization of MPS IIID disease at a cellular level has not been robustly achieved. We used unbiased quantitative proteomics to establish a cellular phenotype for MPS IIID mice. Recombinant human GNS (rhGNS), a variant of which previously demonstrated single dose efficacy in MPS IIID human fibroblasts and in MPS IIID neonatal mice, was used to establish a repeat dosing schedule to treat MPS IIID mice. Adult *Gns* KO mice or heterozygous carriers were treated via intracerebroventricular (ICV) injections and received 3, 30, or 200 μg rhGNS in 4 doses over 2 weeks or vehicle. Twenty-four hours after the final dose, HS in brain and CSF showed dose-dependent reductions, reaching carrier levels in the higher dose groups. Furthermore, the proteomic perturbations that we described were corrected by rhGNS treatment. Next, *Gns* KO or carrier adult mice were treated via ICV and received 3, 30 or 200 μg rhGNS or vehicle once every two weeks (Day 1, 15, 29, 43, 57, 71, 85) and were euthanized on day 91. Following treatment, total HS and MPS IIID-specific HS (GlcNAc6S) showed dose-dependent reductions in brain and CSF and markers of neuroinflammation were substantially reduced. ICV enzyme replacement therapy with rhGNS restores CNS pathology of adult MPS IIID mice even with treatment at 14-day intervals, demonstrating preclinical efficacy for MPS IIID.

## Introduction

Mucopolysaccharidoses (MPS) are a group of lysosomal storage disorders characterized by deficiency in degradation of glycosaminoglycans (GAGs) essential for cellular structure and function in the brain and other tissues [1,2]. MPS III (Sanfilippo syndromes) are a group of MPS diseases that encompass four enzyme deficiencies involved in the degradation of the GAG Heparan Sulfate (HS) [2–5]. The four types of MPS III are MPS IIIA (OMIM: 252900; N-sulfoglucosamine sulfohydrolase, SGSH, deficiency); MPS IIIB (OMIM: 252920; α-N-acetyl glucosaminidase, NAGLU, deficiency); MPS IIIC (OMIM: 252930; heparan sulfate acetyl CoA, α-glucosaminide N-acetyltransferase, HGSNAT, deficiency); and MPS IIID (OMIM: 252940, α-N-acetylglucosamine-6-sulfatase, GNS, deficiency, the rarest MPS III type) [2,6]. MPS IIID (Sanfilippo syndrome type D), as with all MPS III types, leads to accumulation of improperly degraded HS within lysosomes, triggering apoptosis. This abnormal accumulation significantly affects the brain and leads to childhood neurodegeneration, cognitive impairment, dementia, and premature death. Patients also exhibit behavioral issues, poor sleep, seizures, and difficulty walking. Many MPS types clinically present in infancy, MPS IIID, however, begins to clinically affect patients later in childhood [2,3]. Currently, there are few studies looking at the cellular pathology of MPS IIID and there are no approved treatments for MPS IIID.

To address the lack of robust cellular characterization of MPS IIID, we did unbiased quantitative proteomics on 15-week-old adult heterozygous (*Gns + /-,* carrier) and MPS IIID (*Gns-/-*) mice. Gene ontology pathway analysis of this data shows that there is a distinct cellular phenotype of MPS IIID. This data allows us to better understand MPS IIID pathogenesis and gives us an additional measure to use when evaluating treatment efficacy.

Heparan sulfate degradation is a complex process involving many lysosomal enzymes [2]. Enzyme replacement therapy (ERT) is currently being evaluated for multiple MPS III subtypes (ongoing work in our lab) [2]. Our lab has previously shown that myc-tagged recombinant human alpha-N-acetylglucosamine-6-sulfatase (myc-rhGNS) ERT works as a treatment for newborn MPS IIID mice in a single-dose, proof-of-concept study [6]. This study used intracerebroventricular (ICV) administration to deliver ERT directly to the CNS, which will likely also be necessary for patients due to the significant neurological symptoms exhibited by MPS IIID patients. Prior work has shown that ICV administration of therapies is well tolerated in mice and humans for multiple diseases [7–12]. In our initial study, we showed that myc-rhGNS can be successfully produced from Chinese hamster ovary (CHO) cells, that the myc-rhGNS produced from CHO cells has appropriate post-translational modifications, is active *in vitro,* and has efficacy in treating MPS IIID patient fibroblasts. We also delivered the myc-rhGNS to MPS IIID mice with a single ICV dose and this treatment localized to the brain lysosomes, normalized GNS activity, and reduced brain HS.

Herein we describe a treatment for MPS IIID (Sanfillipo D Syndrome) using ERT with rhGNS that builds upon our prior single-dose proof-of-concept study [6]. This prior study in itself was insufficient to support translatable clinical efficacy as repeat doses are needed. Therefore, in the present study we utilized the rhGNS without a myc tag to treat MPS IIID mice with a proof of efficacy repeat dosing regimen and a clinically applicable treatment regimen. We treated heterozygous and MPS IIID mice with vehicle or rhGNS 4 times over 2 weeks. The mice were then euthanized, and their brain tissue and CSF were evaluated for enzyme activity and HS levels. Additionally, in the 2-week cohort, we used proteomic analysis, as mentioned above, to show that there are 56 differentially expressed proteins between the MPS IIID and carrier mice and show that some carrier proteome characteristics are restored after treatment with rhGNS. Using a clinically relevant dosing regimen of one dose every 14 days over a 91-day course (7 doses) we show that HS reduction remains robust, and that glial inflammation, which has been shown to be a significant pathologic feature of MPS IIID mice [4], is significantly reduced. Collectively, the data presented herein represent a meaningful step towards the development of a treatment for MPS IIID.

## Materials and methods

### Mouse models and cannula placement

To eliminate as best as possible, sex as a biological variable, male and female mice were both used with a minimum of 3 mice of each sex per group.

Mouse models as previously described [4,6] were housed at Washington University in St. Louis. A group of *Gns + /-* (carrier) males and females served as controls. They were administered artificial CSF only (vehicle). There were four *Gns-/-* mouse groups each with male and female mice. The groups included a vehicle only group and three treatment groups of 3 μg, 30 μg, and maximum feasible, 200 μg, of rhGNS per dose (Table 1). 200 μg was the maximum based on the concentration we had of rhGNS and the maximum volume that is safe to inject into a mouse ventricle [8]. 3 μg was chosen as the initial dose because it was similar to the dose delivered in our prior study [6]. The other doses were chosen as order of magnitude increases in the base dose. The mice were stereotactically implanted with a sterile guide cannula with dummy cannula (Plastics One, 2.4-mm cut depth, 26 gauge) into the left lateral ventricle, using the following coordinates: 0.3 mm caudal to the bregma, 1 mm lateral to the midline, and 2.4 mm deep as previously described [13,14]. The guide cannula was secured to the skull with cyanoacrylate cement (Scotch Super Glue) to the underside of the cannula and then sealed by dental cement. The implantation was performed under 2% (vol/vol) isoflurane inhalation anesthesia, on an isothermal pad at 37°C. The analgesics buprenorphine and carprofen were administered postoperatively to reduce pain and discomfort. Equal numbers of male and female mice were used in each group except for the 4 doses in 14 days 30 μg per dose treated MPS IIID group, which had 7 males and 3 females. Mice were 15 weeks old at the start of the 4 doses in 14 days treatment and 8 weeks old at the start of the one dose every 14 days for 7 doses treatment.

**Table 1:** Experimental design of mouse treatment groups.

			Treatment Schedule			
			4 Doses over 2 Weeks		7 Doses over 12 Weeks	
	Genotype	μg rhGNS/dose	Male	Female	Male	Female
Numbers of Mice	Carrier (+/-)	0 μg rhGNS	3	3	3	3
	MPS IIID (-/-)	0 μg rhGNS	3	3	3	3
	MPS IIID (-/-)	3 μg rhGNS	3	3	3	3
	MPS IIID (-/-)	30 μg rhGNS	7	3	3	3
	MPS IIID (-/-)	200 μg rhGNS	3	3	3	3

### Vehicle and rhGNS production

Artificial CSF was made with the following recipe: 125 mM NaCl, 22.6 mM NaHCO_3_, 4.4 mM dextrose, 1.2 mM MgSO_4_, 4 mM KCl, 1.4 mM CaCl_2_, 0.75 mM Na_2_HPO_4_; pH 7.2*.* The untagged rhGNS treatment was produced by Waisman Biomanufacturing (Madison, WI) using a CHO cell line derived from a cGMP master cell bank similar to previously described [6]. Purified rhGNS underwent quality control measures including protein concentration via UV spectroscopy (42.5 mg/mL), Purity via Sypro Ruby SDS-PAGE (>95% purity), western blot (dominant band at 90 kDa consistent with reference), sulfatase enzyme activity 2.16x10^4^ nmol/h/mg), residual CHO host cell protein (5003 ppm), and residual DNA by Picogreen (below limit of detection, < 100 ppm).

### Vehicle and rhGNS delivery

The mice were injected with either vehicle or rhGNS (specific activity 3.98 ± 0.14x10^4^ nmol/h/mg) through their implanted cannulas 4 doses over 14 days (treated on days 1, 5, 9, and 14) or one dose every 14 days for 7 total injections (Table 1). To prevent allergic reactions, mice were administered diphenhydramine 5 mg/kg intraperitoneally 15 min prior to treatment/vehicle injection. The mice were anesthetized by 1.5–2.0% (vol/vol) isoflurane inhalation. The dummy canula was removed and an intraventricular internal injector (Plastics One, 2.4-mm cut depth, 33 gauge) was placed into the guide cannula. The internal injector was connected via cannula tubing (PE50, inner and outer diameter 0.58 and 1.27 mm) to a Harvard syringe pump (Harvard Apparatus) to inject 5 μL of rhGNS or 5 μL vehicle over 5 minutes. Twenty-four hours after the last treatment for the 4 doses over 14 days group and six days after the last treatment for the one dose every 14 days group, mice were euthanized, and CSF and brains, divided into hemispheres, were collected for biochemical and microscopic evaluations. In the 14-day study, 24 hours was chosen as the euthanasia date because the goal of that experiment was proof-of-concept for repeat dosing and 24 hours was the most convenient timeframe for euthanasia to answer that question. Additionally, prolonged time from last injection to euthanasia would have likely made enzyme activity assays impossible given the half-life of rhGNS in mice of 1.1 days [6]. Six days was chosen as the euthanasia time for the 12-week study because we sought to understand whether the rhGNS maintained therapeutic effect multiple days after the last injection despite its short half-life.

### Western blots

Samples were loaded on a 4–20% Tris-Glycine Mini-PROTEAN Gels (Bio-Rad) along with Kaleidoscope^TM^ protein standard (Bio-Rad) as a molecular weight marker. Protein bands were transferred onto nitrocellulose membranes using the Trans-Blot Turbo system (Bio-Rad) for western blotting. After blocking with 5% milk in TBST buffer (Tris buffered saline [100 mM Tris, 150 mM NaCl, 2.68 mM KCl, pH 7.4] with 0.1% tween), membranes were probed with either goat anti-GNS antibody (R&D Systems, AF2484), or rabbit anti-GAPDH (CST, Cat# 2118) overnight at 4°C. After washing, membranes were incubated for 2 hours at room temperature with HRP-conjugated secondary antibodies. ECL reagent (WBKLS0500, MilliporeSigma) and ChemiDoc MP Imaging System (Bio-Rad) were used to image the blots.

### Biochemical studies

Tissues were homogenized using a bullet blender (Next Advance, Inc., Troy, NY) with GNS assay buffer containing 0.1% TritonX-100. Homogenized tissues were assayed for GNS and β-hexosaminidase activities. A two-step fluorometric measurement of GNS activity was performed as described previously [15,16] with modifications [6]. A 2.5 µL sample was incubated with 2.5 µL of 10 mM 4-methylumbelliferyl alpha-N-acetylglucosaminide-6-sulfate (4-MUGNS; Toronto Research Chemicals, Toronto, Canada) in the reaction buffer (0.2 M sodium acetate, pH 5.6; 20 mM lead acetate, 0.01% Triton X-100) at 37°C for 4 hours, and the reaction was then stopped by adding 10 µL of phosphate-citrate buffer (0.4 M Na_2_HPO_4_/ 0.2 M citric-acid buffer, pH 4.7). To release the 4-MU fluorophore from the reaction mixture, 5 µL of concentrated rhNAGLU conditioned medium (0.1 mg/mL at specific activity of 2440 nmol/h/mg) was added and incubated for 2 hours at 37°C [14]. Reactions were quenched by adding 50 µL of glycine carbonate buffer (pH 10.5). Fluorescence measurements were obtained using a SpectraMax iD5 Multi-Mode Microplate Reader (Molecular Devices, Sunnyvale, CA) at excitation and emission wavelengths of 360 nm and 450 nm, respectively. One activity unit of GNS was defined as 1 nmol of 4-MU converted substrate per hour (first step) at 37 °C. Protein concentration was determined using Bradford Reagent (Bio-Rad Laboratories, Inc, Irvine, CA). Specific activity was normalized with the protein concentration and presented in units/mg protein. β-hexosaminidase (combined A and B isoforms) was determined by hydrolysis of 4-methylumbelliferyl-N-acetyl-β-glucosaminide (EMD Millipore, Burlington, MA) using 1.25 mM substrate in the incubation mixture for 1 hour [6].

HS levels were determined by the GlycoAnalytics Core (UCSD) as previously described [6,17]. Homogenized mouse brains or mouse CSF samples were digested overnight at 37°C with Pronase (Sigma-Aldrich) in phosphate buffered saline. Supernatant from these samples were collected after centrifuging at 14,000 RPM for 20 min and passed through a DEAE column. The bound GAG was eluted with 2 M NaCl, then desalted on a PD10-size exclusion column, and then lyophilized. The purified and dried GAGs were then dissolved in aniline and mixed with a dimethyl sulfoxide/acetic acid (7:3, v/v) mixture. Reactions in this mixture were done at 65°C for 1 hour and then 37°C for 16 hours. Samples were then dried using a speed vacuum before being analyzed with a glycan reductive isotope labeling (GRIL) LTQ-MS system using a C18 separation column and MS in negative ion mode.

### Microscopy

Immunofluorescence (IF) was done as previously described [4]. Briefly, brain hemispheres were placed in 4% paraformaldehyde for 48 hours followed by transferring to 30% sucrose solution. Brains were sectioned using an HM 430 freezing microtome (Microm International) with a Physitemp BFS 40MOA freezing stage (Physitemp Instruments) to obtain 40-μm-thick coronal sections. A 1 in 6 series of sections were mounted on slides and air-dried. Slides were blocked in 15% normal goat serum (Vector Laboratories) in Tris-buffered saline (TBS) with 2% Triton-X (Alfa Aesar, Ward Hill, MA, USA) followed by incubation in primary antibodies for markers of astrocytes (rabbit anti-GFAP, 1:1000, Dako), microglia (rat anti-mouse CD68, 1:400, Bio-Rad). Sections were rinsed in TBS and incubated in secondary antibody solution (goat anti-rabbit Alexa Fluor 488, 1:400, Invitrogen, and goat anti-rat 546, 1:400, Invitrogen). Slides were rinsed and treated with 1 x TrueBlack (Biotium) before cover slipping with DAPI Fluoromount-G (SouthernBiotech).

Slides for analysis of IF were scanned using a Zeiss Axio Scan.Z1 (Zeiss) at 10X magnification. Scanned images were analyzed using Image-Pro Premier 10 software (Media Cybernetics) using an appropriate threshold that selected the foreground immunoreactivity above background at each region of interest. This threshold was applied as a constant to all images analyzed per group of animals and reagent used to determine the specific area of immunoreactivity for each antigen.

### Proteomics

The protein concentration of each mouse brain lysate was determined using the Bradford method. Fifty μg of proteins digested and desalted using Thermo EasyPep Mini MS Sample Prep Kit (cat# A4006). After determining peptide concentrations by Pierce Quantitative Fluorometric Peptide Assay (cat# 23290), 20 μg peptide from each sample was labeled with TMTpro 16plex Isobaric Label Reagent Set (Thermo, cat# A44522) following the manufacture’s instruction. Labeled samples were combined and dried using vacuum centrifugation. Samples were then separated into 8 fractions using the High pH reversed-phase peptide Fractionation Kit (Thermo, cat# 84868). The fractions were dissolved with 0.1% FA and peptide concentration was determined with Quantitative Colorimetric Peptide Assay (Thermo, cat# 23275).

Tandem mass tag (TMT) labeling LC-MS/MS experiments were performed using an EASY-nLC 1000 connected to an Orbitrap Eclipse Tribrid mass spectrometer. 0.5 μg of each fraction was loaded onto an Aurora UHPLC Column and separated over 131 min at a flow rate of 0.4 μL/min with the following gradient: 2–6% Solvent B (2 min), 6–22% B (78 min), 22–50% B (40 min), 50–95% B (1 min), and 95% B (10 min). Solvent A consisted of 97.8% H_2_O, 2% ACN, and 0.2% formic acid, and solvent B consisted of 19.8% H_2_O, 80% ACN, and 0.2% formic acid. An MS1 scan was acquired in the Orbitrap at 120k resolution with a scan range of 400–1600 m/z. The AGC target was 1 x 10^6^, and the maximum injection time was 50 ms. Dynamic exclusion was set to exclude features after 1 time for 60 s with a 10-ppm mass tolerance. MS2 scans were acquired with CID activation type with the IonTrap. The isolation window was 0.7 m/z, the collision energy was 35%, the maximum injection time was 35 ms and the AGC target was 1 x 10^4^. MS3 scans were acquired with HCD activation type in the Orbitrap at 60k resolution with a scan range of 100–500 m/z. The isolation window was 0.7 m/z, the collision energy was 65%, the maximum injection time was 118 ms and the AGC target was 2.5 x 10^5^. Ion source settings were as follows: ion source type, NSI; spray voltage, 2300 V; ion transfer tube temperature, 300°C. System control and data collection were performed by Xcalibur software.

TMT labelling was employed to evaluate the proteomic changes caused by MPS IIID. Specifically, we prepared three independent biological carrier, MPS IIID, and 200 μg ERT treated MPS IIID (ERT) mouse brain samples. The TMT labeled samples were pooled and further fractionated into 8 fractions and analyzed with an RTS-SPS-MS3 method. A total of 5214 proteins were identified and quantified across all 9 samples (S1 Data). We identiﬁed 555 proteins that demonstrated signiﬁcantly diﬀerent quantities (p < 0.05) between the MPS IIID and heterozygote samples. Among them, a set of 56 proteins with log2 (fold change) >0.5 (up-regulated) or <−0.5 (down-regulated) was classified as the ﬁnal diﬀerentially expressed proteins (DEPs).

### Statistics and software

Biochemical results were plotted with Prism 10 (GraphPad, Inc., La Jolla, CA) and analyzed for significance with one-way ANOVA with multiple comparisons with a Dunnett correction or with two-way ANOVA with multiple comparisons with a Tukey correction. IF results were analyzed via threshold analysis as above and plotted with Prism 10. Significance was determined with two-way ANOVA with multiple comparisons with a Tukey correction. The proteomic data processing was performed through Proteome Discoverer 2.4 (Thermo Fisher) using Uniprot canine database and the SequestHT with Percolator validation. Normalization was performed relative to the total peptide amount. DEPs were tested for enrichment analysis using g:Profiler (2023 update) [18]. Enrichment analysis looked at KEGG [19] terms and Gene Ontology [20,21] terms using *Mus musculus* as the organism and the Benjamini-Hochberg FDR correction and plotted with Prism 10; PCA and individual heatmap generated with ClustVis web tool [22]. For bar graphs of biochemical and IF data throughout, results are the mean of the data and error bars are standard deviation.

### Study approval and ethics statement

Animal experiments were approved by the Institutional Animal Care and Use Committee at Washington University School of Medicine in St. Louis, which is accredited by the Association for Assessment and Accreditation of Laboratory Animal Care (AAALAC).

## Results

### Proteomic characterization of MPS IIID mice

**Fig 1:**
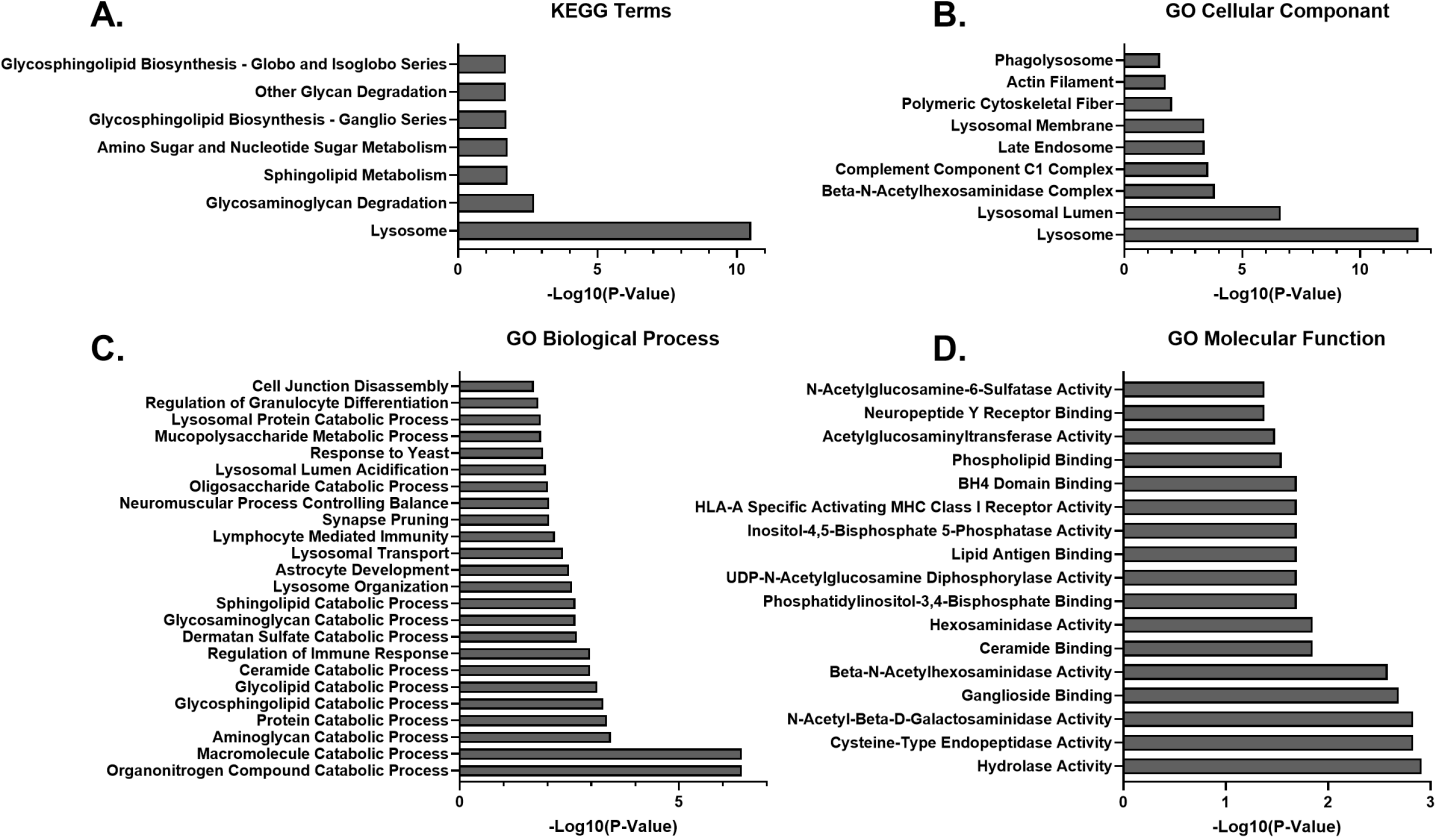
Gene Ontology Analysis of MPS IIID Mice Compared to Carrier Mice. **A)** KEGG terms that are significantly different based on the 56 differentially expressed proteins between the MPS IIID mice and carrier mice. **B)** GO Cellular Component terms that are significantly different based on the 56 differentially expressed proteins between the MPS IIID mice and carrier mice. **C)** GO Biological Process terms that are significantly different based on the 56 differentially expressed proteins between the MPS IIID mice and carrier mice. **D)** GO Molecular Function terms that are significantly different based on the 56 differentially expressed proteins between the MPS IIID mice and carrier mice.

Using TMT labeled proteomic analysis, we investigated what pathways were affected by protein expression changes. Gene Ontology (GO) and KEGG analysis [19–21] of the proteome data for 15-week-old MPS IIID vs carrier mice showed significance for changes in pathways related to lysosomal regulation, macromolecule catabolism, and immune function (Fig 1 and S1 Data). Proteins important to lysosomal regulation and macromolecule catabolism were significantly altered in the MPS IIID mice relative to carrier consistent with MPS IIID being a lysosomal storage disease. These changes were similar across the different GO categories (molecular function, biological process, and cellular component). For example, the lysosome as a cellular component was significantly changed, which correlates with the many catabolic processes that were changed in the biological process terms, which, again, is consistent with hydrolase activity being a changed molecular function. The significant GO terms were similar to the significant KEGG terms.

### Biochemical analysis after two-week repeated rhGNS ICV treatment

**Fig 2:**
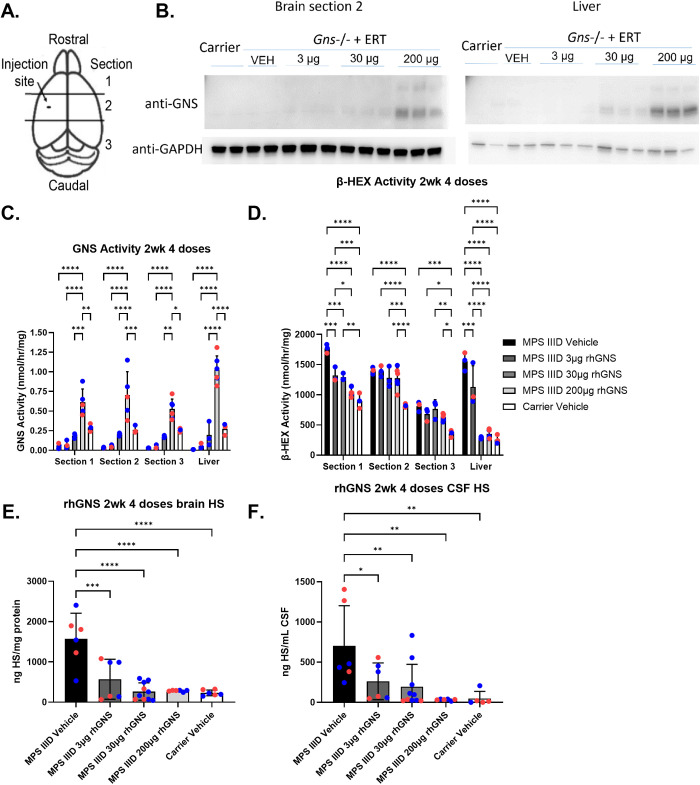
Biochemical Analysis of Mice Treated with rhGNS for 2 Weeks. **A)** Diagram of a mouse brain sectioned into 3 sections from rostral to caudal with treatment injection site marked. Brains were harvested 6 days after the final rhGNS dose. Diagram modified from [6]. **B)** Western blots showing GNS protein levels in brains (section 2) and livers of mice treated with increasing doses of rhGNS for 4 doses over 2 weeks. GAPDH levels shown as a loading control. **C)** GNS activity in brains (sectioned into 3 sections from rostral to caudal) and liver. **D)** β-HEX activity in brains (sectioned into 3 sections from rostral to caudal) and liver. **E)** HS levels in the brain of mice from section 1. **F)** HS levels in the CSF of mice. In C-F, blue data points are male and red data points are female. P values: * < 0.05, ** < 0.01, *** < 0.001, **** < 0.0001.

Our prior proof-of-concept study into myc-rhGNS treatment of MPS IIID used a single dose in newborn mice without implanted ICV cannulas. Therefore, we aimed to first test the tolerance of the mice to the implanted ICV cannula and to repeat dosing of untagged rhGNS while also assessing treatment efficacy. To our knowledge there is no reported difference in MPS IIID pathology between male and female mice [4], however, we included at least three male and three female mice per group and analyzed for sex differences throughout the study where possible (Table 1). Adult 15-week-old carrier and MPS IIID mice were implanted with ICV cannulas and treated with vehicle (artificial CSF) or increasing doses (3 μg, 30 μg, or 200 μg) of rhGNS for 4 doses over 14 days. The mice successfully tolerated the surgery and survived to the end of the study without early death or weight loss. Following completion of the treatment regimen, the mice were euthanized 24 hours after their last dose, and their brains and livers dissected, sectioned (for brains as in Fig 2A), and analyzed for GNS concentration via western blot, GNS activity, and beta-hexosaminidase activity; and their brains and CSF were analyzed for HS levels. The western blot showed an increasing GNS signal in the brains and livers with increasing dose of rhGNS delivered (Fig 2B). GNS activity similarly increased in the brains and livers of the mice with increasing dose of rhGNS delivered, and this was true in all brain slices indicating good parenchymal penetration (Fig 2C). The rhGNS is seen in the liver because CSF is constantly being secreted from the CNS into the blood and lymphatic systems so its contents can be metabolized and excreted from the body (CSF physiology reviewed in [23]). The 3 brain sections and the liver were then tested for beta-hexosaminidase (β-HEX, combined A and B isoforms, lysosomal enzymes that accumulate in lysosomal storage disorders) activity. The treated mice showed decreases in β-HEX, also in a dose-dependent manner with significant decreases in β-HEX seen in brain section 1 (Fig 2D). The mice showed a dose-responsive decrease in the levels of HS in the CSF and brain with the highest dose resulting in levels essentially identical to the carrier mice and significantly decreased compared to the vehicle treated MPS IIID mice (Figs 2E and 2F). There were no significant sex differences between the mice in these analyses. These results, collectively, indicate that rhGNS is getting into the brain and distributing throughout the parenchyma, is active and effective, and that very low levels of rhGNS (below limit of detection on western blot) can have a significant impact on disease biochemistry.

### Proteomic characterization after repeated ICV rhGNS treatment

**Fig 3:**
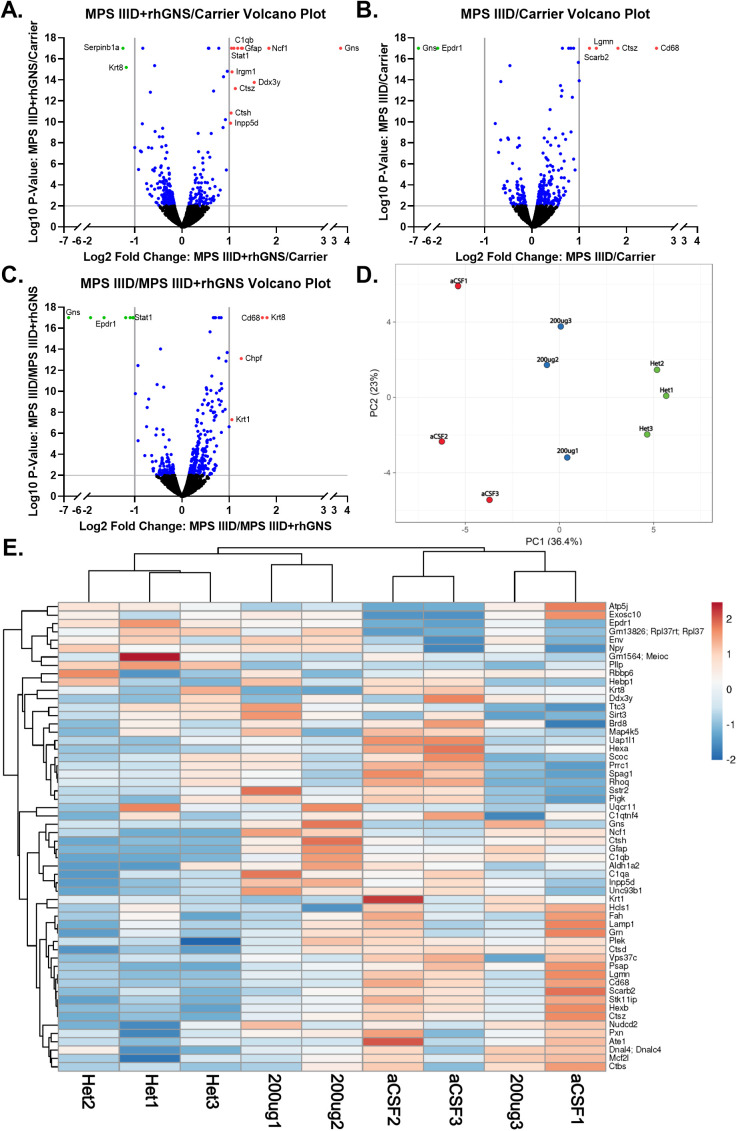
Proteomic Analysis of Mice Treated with rhGNS for 2 Weeks. **A)** Volcano plot of protein expression of MPS IIID mice treated with rhGNS relative to carrier mice. **B)** Volcano plot of protein expression of MPS IIID mice relative to carrier mice. **C)** Volcano plot of protein expression of MPS IIID mice relative to MPS IIID mice treated with rhGNS. **D)** PCA plot of the individual mice from all groups showing the distribution of the mice due to the relative abundances of the 56 proteins that are differentially expressed between the MPS IIID mice and the carrier mice. **E)** Heatmap showing the differential expression of the 56 proteins that are differentially expressed between the MPS IIID mice (treated and untreated) and the carrier mice. In D and E, “Het” labels are short for heterozygous and have the same meaning as carrier as presented elsewhere in this manuscript.

To see if treatment with rhGNS corrects the proteomic perturbations described above, we used brain lysates from the vehicle treated MPS IIID and carrier mice and the 200 μg rhGNS dose from the 15-week-old 4 doses in 2 weeks treatment regimen MPS IIID mice to run TMT labeled proteomic analysis of their brains. While 30 μg dosing showed robust biochemical correction in our mice, the 200 μg group was chosen for proteomic analysis because it was slightly more efficacious than the 30 μg group while still being safe; and because there is evidence that in prior clinical trials of ERT for LSDs that using the highest possible dose leads to better patient outcomes (dose escalation/determination for LSDs in clinical trials reviewed in [24,25]). Differentially expressed proteins of the different groups of mice relative to each other group were determined and visualized on volcano plots (Figs 3A-3C). The limits for describing differential expression used in the subsequent analyses are less strict than what is visualized in the volcano plots (details in methods), which only highlight the most significant differentially expressed proteins. Unsurprisingly, GNS was significantly elevated in the carrier group relative to the MPS IIID group and CD68 was significantly elevated in the MPS IIID group relative to the carrier group (Figs 3B and 3E), confirming our disease phenotype is due to appropriate gene knockout and correlating with our immunofluorescence (IF) data (see below). Additionally, lysosomal proteins Hexosaminidase A, Hexosaminidase B, and LAMP1 were all elevated in MPS IIID mice relative to carrier mice and there were several proteins that were downregulated in MPS IIID mice relative to carriers (Figs 3B and 3E). Treatment with rhGNS corrected many of these perturbations as seen by comparing the volcano plots and heatmap of the MPS IIID mice with the treated MPS IIID mice and by comparing the MPS IIID treated mice with the carrier mice (Figs 3A, 3C, and 3E). Using the 56 differentially expressed proteins between the MPS IIID mice and the carrier mice, the relative abundances of these proteins from the individual mice were analyzed via a PCA plot and heatmap. The PCA plot showed that the groups of mice are distinct from each other indicating that the treated MPS IIID mice underwent some significant proteomic changes because of the treatment (Fig 3D). The heatmap shows that of the 3 treated MPS IIID mice, 2 cluster with the Carrier mice and one clusters with the MPS IIID mice, results consistent with the PCA plot while also indicating that there are likely additional variables that impact individual response to treatment. Interestingly, GFAP was elevated in the rhGNS treated MPS IIID mice relative to the carrier mice and relative to the MPS IIID vehicle treated mice, which is discordant with the IF data (see below). The proteomic data encompasses the GFAP of the entire brain, while the IF data looked at specific regions of slices of the brain, and the treatment regimen for the IF data and the proteomic data were different, which could explain the discordance. Other than GFAP, the proteomic data were validated by the biochemical and histologic data presented herein.

### Immunofluorescence after long-term ICV rhGNS treatment

We then aimed to test a more clinically relevant dosing schedule for rhGNS. Adult 8-week-old carrier and MPS IIID mice were again implanted with ICV cannulas and were treated with single doses of vehicle or rhGNS (3 μg, 30 μg, or 200 μg) every 14 days for 7 doses and euthanized 6 days following their final dose. One dose every 14 days was chosen because it is clinically feasible (asking patients to go to an infusion center more than every other week would likely decrease compliance) and is likely to remain efficacious based on our prior work [6]. These mice tolerated the treatment well without premature death or significant weight loss. The brains were collected and separated into hemispheres, one for HS analysis and the other for IF analysis.

**Fig 4:**
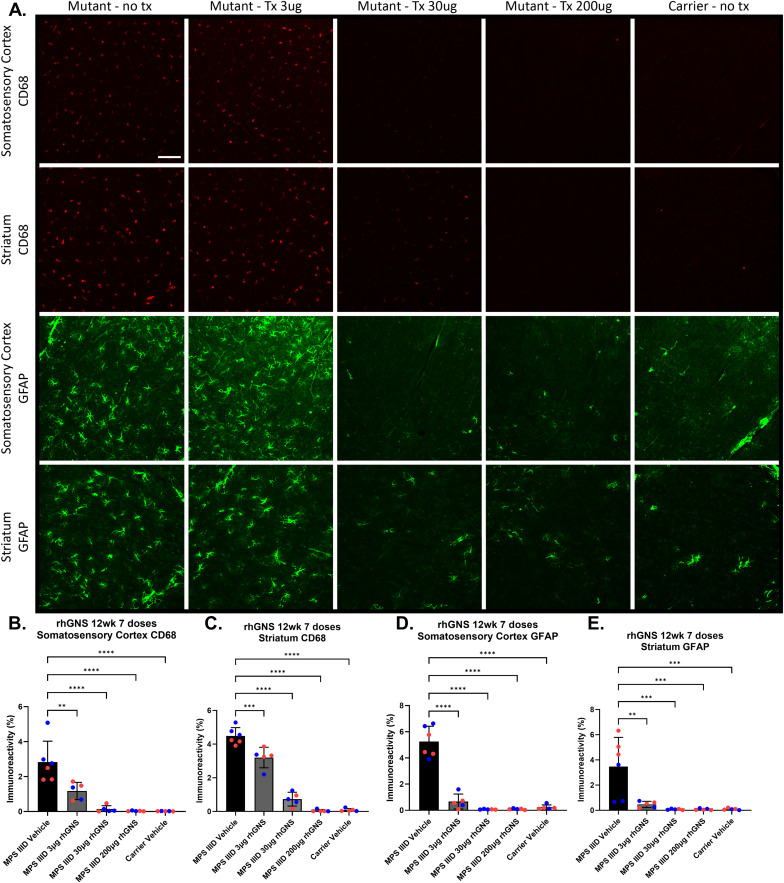
Immunofluorescence Analysis of Mice Treated with rhGNS for 12 Weeks. **A)** representative CD68 and GFAP IF images from the somatosensory cortex and striatum from brains from each treatment group. Images are at 20X magnification and scale bar represents 100 μm. **B)** Quantification of IF signal from somatosensory cortex CD68 data. **C)** Quantification of IF signal from striatum CD68 data. **D)** Quantification of IF signal from somatosensory cortex GFAP data. **E)** Quantification of IF signal from striatum GFAP data. In the quantification data, blue data points are male and red data points are female. P values: ** < 0.01, *** < 0.001, **** < 0.0001.

MPS IIID mice display significant and progressive glial inflammation [4]. We examined the somatosensory cortex and the striatum from the mice treated every 14 days for 7 doses using IF for GFAP and CD68 (markers of astrocytes and activated microglia, respectively). The mice treated with rhGNS showed dose-dependent reductions in CD68 and GFAP (Fig 4), with the 200 μg treatment dose lowering the levels of gliosis to carrier levels in these brain regions. Gliosis is a significant driver of pathology in the MPS IIID mice and has been noted in MPS IIID patient autopsies as well [4]. Our results demonstrate that correction of the enzyme deficiency in MPS IIID mice with rhGNS ERT corrects pathogenic gliosis. There was a significant difference between the GFAP levels of male and female mice in the striatum of the MPS IIID vehicle treated mice. In all other groups of mice for the striatum and in all groups for the somatosensory cortex there were no sex differences between the mice.

### Heparan sulfate analysis after long-term ICV rhGNS treatment

**Fig 5:**
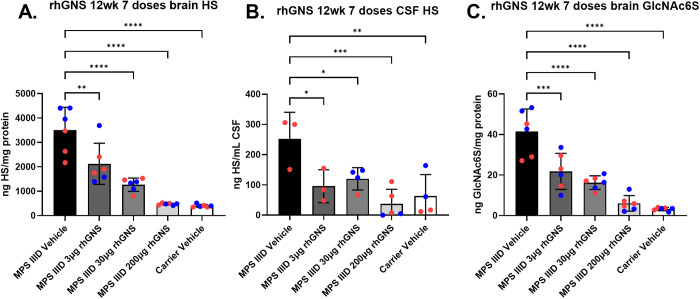
Heparan Sulfate Levels in Mice CNS after 12 Week Treatment with rhGNS. **A)** Heparan Sulfate (HS) levels in the brain of mice treated with different doses of rhGNS for 7 doses over 12 weeks. Mice were euthanized 6 days after their final dose. **B)** HS levels in the CSF of mice treated with increasing doses of rhGNS for 7 doses over 12 weeks. **C)** GlcNAc6S levels in the brain of mice. Blue data points are male and red data points are female. P values: * < 0.05, ** < 0.01, *** < 0.001, **** < 0.0001.

Brain hemispheres and CSF samples from the long-term treated mice were collected for HS analysis. The mice exhibited a dose-responsive decrease in the levels of HS in the CSF and brain (Fig 5A and 5B). This dose-dependent effect was also illustrated with the reduction of N-acetyl-D-glucosamine 6-sulfate (GlcNAc6S; a GAG elevated specifically in MPS IIID) in brain, showing that rhGNS is correcting the specific enzyme deficiency in MPS IIID (Fig 5C). In the brain, the 200 μg treatment group had HS and GlcNAc6S levels similar to carrier levels. CSF samples were not able to be obtained for all mice due to technical difficulties during CSF harvest and therefore sex difference analysis was not done on the CSF HS levels for this treatment regimen due to limited sample numbers. CSF HS was chosen as a biological sample over urine or blood HS because CSF HS is specifically a marker of CNS HS pathology whereas blood or urine HS would be reflective of CNS and peripheral HS pathology [5]. There were otherwise no significant sex differences between the mice in these analyses.

## Discussion

MPS IIID, while rare, is a devastating disease with significant morbidity and mortality that has no current treatments. Herein, we expanded on our prior work by presenting data further characterizing the cellular pathogenesis of MPS IIID mice and showing that ICV delivered rhGNS ERT to adult mice abrogates this pathology and treatment is well tolerated. Our prior study showed that CHO expressed myc-rhGNS is highly active and purifiable, and when delivered via ICV to newborn MPS IIID mice, shows a significant reduction in pathogenic HS deposits in the brain [6]. This study was valuable in establishing a treatment option for MPS IIID, however, it did not establish a clinically applicable dosing regimen and did little to describe the effects of treatment on disease pathology. In the present study, we showed through proteomic analysis that MPS IIID mice exhibit perturbations in several crucial cellular pathways. We also showed that rhGNS delivered repeatedly via an ICV method degrades the pathologically accumulated HS from the brain of adult mice and that clearing these accumulations corrects cellular proteome disruptions. Finally, we showed that with a clinically relevant dosing schedule, rhGNS ICV treatment leads to significant reductions in pathogenic HS and corrects neuroinflammation.

We did not evaluate behavior or survival in the mice in this study. Survival improvement would not have been feasible to measure in our MPS IIID mice because they have a normal lifespan consistent with the background strain. Behavioral phenotypes of MPS III A and B mice have been relatively well studied [26–32], behavior of MPS IIID mice have not been well studied [33], with no behavioral studies yet performed in the mouse model employed here. Therefore, complete validation of a behavioral phenotype in our mouse model is beyond the scope of this study and pursuing this data would delay bringing an otherwise safe and biochemically effective treatment to the clinic. Furthermore, expert consensus is that CSF HS analysis, as performed in this study, is a significantly robust biomarker of disease and can be used as an early clinical endpoint for neuronopathic MPS disease [5]. Phenotypic/behavioral changes in mice with genetic disease can be a beneficial tool in evaluating treatment efficacy, however, these changes (if present at all) do not always correlate well to human disease [34]. Before rhGNS can be fully approved for clinical use, we acknowledge that phenotypic/behavioral benefit will need to be demonstrated in humans. We believe that this would be best achieved in a clinical trial extension following initial validation of biochemical benefit in patients.

Both groups of mice in our study were aged to adulthood prior to treatment initiation. This could explain why the proteomics of the treated MPS IIID mice did not show a complete alignment with the heterozygous proteome. As with other LSDs, especially those with neurological sequelae, early treatment of MPS IIID will likely lead to better clinical results. We elected to use adult mice in our study for two reasons. The first is that it is challenging to do repeat ICV dosing in neonatal mice. The second is that MPS IIID is a rare disease even amongst other MPS diseases. Therefore, it is likely that a clinical trial will necessitate enrollment of older patients and showing preclinical benefit in adult mice is more likely to translate to some success in older patients than a therapy that only works well when initiated in newborn mice.

In the present study, we performed an initial characterization of the proteome and cellular pathway perturbations in MPS IIID mice, which serves as a surrogate phenotypic endpoint in the absence of behavioral data. Many of the differences between the MPS IIID mice and the carriers were expected given that MPS IIID is a lysosomal storage disease, including the many catabolic processes that appear changed and the localization of changed proteins to the lysosome. Interestingly, microglia function appears to be significantly dysregulated in the MPS IIID mice. In addition to CD68 being upregulated (Figs 3 and 4), which indicates increased microglial activation in response to stress, other functions of microglia also appear to be altered (Figs 1 and 3). Microglia are important to the health of the CNS for many reasons [35,36] including CNS regulation through synapse pruning, a process which involves the C1q complement component. Microglia activation also involves significant cytoskeleton remodeling. Our proteomic data showed GO terms involving C1q, synaptic pruning, and actin filaments were all significantly changed in MPS IIID mice relative to carriers. All the proteins contributing to these changes were upregulated (S1 Data). While collectively these data indicate significant microglial pathology in MPS IIID mice, it is unclear from these data whether the microglia are responding to neuronal pathology leading to activation and inflammation, are undergoing their own pathology that then leads to neuronal damage through the induced inflammation, or some combination of these scenarios. These proteomic data are an important first step in better understanding the cellular pathology of MPS IIID, but they will require more analysis and follow-up, hypothesis driven, experiments to fully understand the implicated disease pathways. Importantly, the proteomic data presented here showed that many of the protein disruptions were corrected by treatment with rhGNS and much of the proteomic data was congruent with the biochemical data.

It is now well agreed within the MPS clinical and research community that HS levels in the CNS are a reliable marker of the level of CNS disease [5]. For this reason, CSF collection was used throughout our manuscript instead of blood or urine HS collection, which would not be specific for CNS disease. Because of this consensus and because the long treatment regimen was still done on adult mice, we did not feel that repeating proteomics on the 7 doses over 12 weeks treated mice would have added substantial conclusions to our study. It is a limitation to not have this data but likely a minor one. We believe that the HS and IF data is sufficient to show that rhGNS remains effective on the clinically feasible treatment regimen of one dose every two weeks.

Sex is a modifier of many diseases including some lysosomal diseases [37,38]. We are unaware of any significant sex discrepancies in patients or mice with MPS IIID, however, we tried to ensure that equal numbers of male and female mice were used in our experiments to avoid missing any unforeseen sex differences. Our prior work indicated that gliosis in MPS IIID mice does not change based on sex [4]. In our study, however, the GFAP levels in the striatum of the MPS IIID vehicle treated mice were significantly higher in the female mice than the males. It is unclear why there is a discrepancy between these two studies. Further work should continue to utilize equal male and female mice to ensure that there is no sex difference in MPS IIID or to confirm our observation of increased astrogliosis in the striatum of female mice.

This study utilized ICV injections through an implanted catheter to deliver ERT to the CNS. ICV is safe in humans and mice and is an effective way to get therapy into the CNS [7,8,39]. There have been concerns about ICV effectiveness in larger animals due to the mechanics of CSF flow and the difficulty of parenchymal penetration [40,41]. We have shown in prior studies of canine intrathecal (IT) ERT administration for MPS I that there is good brain parenchyma penetration [42,43] and our prior study of myc-rhGNS demonstrated good distribution throughout murine brains. The efficacy of direct CNS delivery via ICV or IT is likely dependent on the individual therapy being delivered and the disease phenotype. Despite ICV and IT injections being invasive, requiring significant expertise to do, having risks of complications, and having possible challenges in patient compliance, these methods are now a staple of clinical CNS drug delivery [44]. ICV ERT treatments for genetic disease will, by necessity, be lifelong, which is a substantial burden on patients despite the current level of routineness for ICV in the clinic. ERT delivery by ICV is the most direct way to treat neuronopathic MPS disease, however, as new treatment modalities are developed (discussed below), the hope is that treatment becomes less of a burden to patients and ICV injections are either not necessary or less frequent.

There are many active areas of research aiming to improve administration of therapies to the CNS including using ERT attached to antibodies targeting the transferrin receptor, intranasal delivery, and blood brain barrier disruption with hyperosmolar agents or pulsed ultrasound, among others [1,45–51]. However, none of these methods are currently normal clinical practice for CNS drug delivery and are likely several years from being as effective as ICV or IT [45,52]. There is evidence that in neurodegenerative and neuroinflammatory diseases, the blood brain barrier is leaky and peripherally administered therapy can get into the CNS without additional measures [53,54]. Even with a leaky blood brain barrier, peripheral doses of ERT would likely not reach significantly high doses in the CNS. Future work will be necessary to reevaluate rhGNS delivery to the CNS using one of the less invasive methods, but currently ICV injection is the best method to get an ERT therapy to the CNS of MPS IIID patients.

Gene therapy (both *in vivo* [vectors delivered to the patient directly] and *ex vivo* [autologous hematopoietic stem cell transplant following gene therapy given to the cells]) is another option for treating MPS IIID that has several benefits over ERT including fewer required doses, better integration of normal post-translational modifications, and possible better targeting to disease relevant tissues [55]. Discussions on the state of gene therapy for genetic diseases is reviewed elsewhere [56–58]. ERT has advantages over gene therapy including ability to titrate dose and avoidance of pre-existing anti-capsid antibodies, however ERT, like all biologic therapy, includes risk of developing an immune response to therapy [55,59]. ERT can carry an important role then of bridging patients until gene therapy is developed because it is often quicker to develop into a clinical therapy and there is no guarantee that gene therapies for certain disease will ever make it to the clinic. Indeed, the clinical success of several ERT regimens for many diseases including MPS I (laronidase), MPS II (idursulfase), MPS VI (galsulfase), Pompe disease (alglucosidase alfa), Gaucher disease (imiglucerase), Fabry disease (agalsidase β), and Batten disease (cerliponase alfa) indicate that rhGNS will likely have a significant clinical role even if further research into gene therapy is pursued [42,43,60,61].

## Conclusions

This study expands on our prior work with myc-rhGNS to establish a viable ERT treatment for MPS IIID; and provides further evidence that significant cellular disruptions in the brains of MPS IIID mice exist and that rhGNS corrects this pathology when delivered directly to the CNS in adult animals. This preclinical evidence along with expert consensus that CSF HS is a sufficient biomarker to use as a clinical endpoint support advancing research on rhGNS to higher animal or human trials to bring ERT for MPS IIID to the clinic.

## Supporting information

S1 data This file contains Supplementary Figures and Total Profiler data details. (XLSX)

## References

[1] Scarpa M, Orchard PJ, Schulz A, Dickson PI, Haskins ME, Escolar ML, et al. Treatment of brain disease in the mucopolysaccharidoses. Mol Genet Metab. 2017;122:25–34. doi: 10.1016/j.ymgme.2017.10.007 29153844

[2] Benetó N, Vilageliu L, Grinberg D, Canals I. Sanfilippo syndrome: molecular basis, disease models and therapeutic approaches. Int J Mol Sci. 2020;21(21):7819. doi: 10.3390/ijms21217819 33105639 PMC7659972

[3] Pearse Y, Clarke D, Kan S-H, Le SQ, Sanghez V, Luzzi A, et al. Brain transplantation of genetically corrected Sanfilippo type B neural stem cells induces partial cross-correction of the disease. Mol Ther Methods Clin Dev. 2022;27:452–63. doi: 10.1016/j.omtm.2022.10.013 36419468 PMC9672419

[4] Takahashi K, Le SQ, Kan S-H, Jansen MJ, Dickson PI, Cooper JD. Neuropathology of murine Sanfilippo D syndrome. Mol Genet Metab. 2021;134(4):323–9. doi: 10.1016/j.ymgme.2021.11.010 34844863

[5] Muenzer J, Ho C, Lau H, Dant M, Fuller M, Boulos N, et al. Community consensus for Heparan sulfate as a biomarker to support accelerated approval in Neuronopathic Mucopolysaccharidoses. Mol Genet Metab. 2024;142(4):108535. doi: 10.1016/j.ymgme.2024.108535 39018614

[6] Wang F, Moen DR, Sauni C, Kan S-H, Li S, Le SQ, et al. Enzyme replacement therapy for mucopolysaccharidosis IIID using recombinant human α-N-Acetylglucosamine-6-Sulfatase in neonatal mice. Mol Pharm. 2021;18(1):214–27. doi: 10.1021/acs.molpharmaceut.0c00831 33320673 PMC8362844

[7] Cohen-Pfeffer JL, Gururangan S, Lester T, Lim DA, Shaywitz AJ, Westphal M, et al. Intracerebroventricular delivery as a safe, long-term route of drug administration. Pediatr Neurol. 2017;67:23–35. doi: 10.1016/j.pediatrneurol.2016.10.022 28089765

[8] Slavc I, Cohen-Pfeffer JL, Gururangan S, Krauser J, Lim DA, Maldaun M, et al. Best practices for the use of intracerebroventricular drug delivery devices. Mol Genet Metab. 2018;124(3):184–8. doi: 10.1016/j.ymgme.2018.05.003 29793829

[9] Lewis G, Morrill AM, Conway-Allen SL, Kim B. Review of cerliponase alfa: recombinant human enzyme replacement therapy for late-infantile neuronal ceroid lipofuscinosis type 2. J Child Neurol. 2020;35(5):348–53. doi: 10.1177/0883073819895694 31884868

[10] Zhou Z, Austin GL, Shaffer R, Armstrong DD, Gentry MS. Antibody-mediated enzyme therapeutics and applications in glycogen storage diseases. Trends Mol Med. 2019;25(12):1094–109. doi: 10.1016/j.molmed.2019.08.005 31522955 PMC6889062

[11] Austin GL, Simmons ZR, Klier JE, Rondon A, Hodges BL, Shaffer R, et al. Central nervous system delivery and biodistribution analysis of an antibody-enzyme fusion for the treatment of lafora disease. Mol Pharm. 2019;16(9):3791–801. doi: 10.1021/acs.molpharmaceut.9b00396 31329461 PMC7189208

[12] Brewer MK, Uittenbogaard A, Austin GL, Segvich DM, DePaoli-Roach A, Roach PJ, et al. Targeting pathogenic lafora bodies in lafora disease using an antibody-enzyme fusion. Cell Metab. 2019;30(4): 689–705. doi: 10.1016/j.cmet.2019.07.002 31353261 PMC6774808

[13] Kan S-H, Elsharkawi I, Le SQ, Prill H, Mangini L, Cooper JD, et al. Biochemical evaluation of intracerebroventricular rhNAGLU-IGF2 enzyme replacement therapy in neonatal mice with Sanfilippo B syndrome. Mol Genet Metab. 2021;133(2):185–92. doi: 10.1016/j.ymgme.2021.03.013 33839004 PMC8195848

[14] Kan S-H, Aoyagi-Scharber M, Le SQ, Vincelette J, Ohmi K, Bullens S, et al. Delivery of an enzyme-IGFII fusion protein to the mouse brain is therapeutic for mucopolysaccharidosis type IIIB. Proc Natl Acad Sci U S A. 2014;111(41):14870–5. doi: 10.1073/pnas.1416660111 25267636 PMC4205671

[15] Karpova EA, Voznyi YA, Keulemans JLM, Hoogeveen AT, Winchester B, Tsvetkova IV, et al. A fluorimetric enzyme assay for the diagnosis of Sanfilippo disease type A (MPS IIIA). J Inherit Metab Dis. 1996;19(3):278–85. doi: 10.1007/BF01799255 8803769

[16] He W, Voznyi YV, Boer AM, Kleijer WJ, van Diggelen OP. A fluorimetric enzyme assay for the diagnosis of Sanfilippo disease type D (MPS IIID). J Inherit Metab Dis. 1993;16(6):935–41. doi: 10.1007/BF00711508 8127069

[17] Lawrence R, Olson SK, Steele RE, Wang L, Warrior R, Cummings RD, et al. Evolutionary differences in glycosaminoglycan fine structure detected by quantitative glycan reductive isotope labeling. J Biol Chem. 2008;283(48):33674–84. doi: 10.1074/jbc.M804288200 18818196 PMC2586254

[18] Kolberg L, Raudvere U, Kuzmin I, Adler P, Vilo J, Peterson H. g:Profiler-interoperable web service for functional enrichment analysis and gene identifier mapping (2023 update). Nucleic Acids Res. 2023;51(W1):W207–12. doi: 10.1093/nar/gkad347 37144459 PMC10320099

[19] Kanehisa M, Goto S. KEGG: kyoto encyclopedia of genes and genomes. Nucleic Acids Res. 2000;28(1):27–30. doi: 10.1093/nar/28.1.27 10592173 PMC102409

[20] Ashburner M, Ball CA, Blake JA, Botstein D, Butler H, Cherry JM, et al. Gene Ontology: tool for the unification of biology. Nat Genet. 2000;25(1):25–9. doi: 10.1038/75556 10802651 PMC3037419

[21] Consortium TGO, Aleksander SA, Balhoff J, Carbon S, Cherry JM, Drabkin HJ, Ebert D, et al. The Gene Ontology knowledgebase in 2023. Genetics. 2023;224(1). doi: 10.1093/genetics/iyad031 PMC10158837 36866529

[22] Metsalu T, Vilo J. ClustVis: a web tool for visualizing clustering of multivariate data using Principal Component Analysis and heatmap. Nucleic Acids Res. 2015;43(W1):W566–70. doi: 10.1093/nar/gkv468 PMC4489295 25969447

[23] Sakka L, Coll G, Chazal J. Anatomy and physiology of cerebrospinal fluid. Eur Ann Otorhinolaryngol Head Neck Dis. 2011;128(6):309–16. doi: 10.1016/j.anorl.2011.03.002 22100360

[24] Hon YY, Wang J, Abodakpi H, Balakrishnan A, Pacanowski M, Chakder S, et al. Dose selection for biological enzyme replacement therapy indicated for inborn errors of metabolism. Clin Transl Sci. 2023;16(12):2438–57. doi: 10.1111/cts.13652 37735717 PMC10719471

[25] Ellison S, Parker H, Bigger B. Advances in therapies for neurological lysosomal storage disorders. J Inherit Metab Dis. 2023;46(5):874–905. doi: 10.1002/jimd.12615 37078180

[26] Kan S-H, Le SQ, Bui QD, Benedict B, Cushman J, Sands MS, et al. Behavioral deficits and cholinergic pathway abnormalities in male Sanfilippo B mice. Behav Brain Res. 2016;312:265–71. doi: 10.1016/j.bbr.2016.06.023 27340089 PMC4970944

[27] Lau AA, Crawley AC, Hopwood JJ, Hemsley KM. Open field locomotor activity and anxiety-related behaviors in mucopolysaccharidosis type IIIA mice. Behav Brain Res. 2008;191(1):130–6. doi: 10.1016/j.bbr.2008.03.024 18453006

[28] Crawley AC, Gliddon BL, Auclair D, Brodie SL, Hirte C, King BM, et al. Characterization of a C57BL/6 congenic mouse strain of mucopolysaccharidosis type IIIA. Brain Res. 2006;1104(1):1–17. doi: 10.1016/j.brainres.2006.05.079 16828069

[29] Heldermon CD, Hennig AK, Ohlemiller KK, Ogilvie JM, Herzog ED, Breidenbach A, et al. Development of sensory, motor and behavioral deficits in the murine model of Sanfilippo syndrome type B. PLoS One. 2007;2(8):e772. doi: 10.1371/journal.pone.0000772 17712420 PMC1945015

[30] McCullough KB, Titus A, Reardon K, Conyers S, Dougherty JD, Ge X, et al. Characterization of early markers of disease in the mouse model of mucopolysaccharidosis IIIB. J Neurodev Disord. 2024;16(1):16. doi: 10.1186/s11689-024-09534-z 38632525 PMC11022360

[31] Pericleous K, McIntyre C, Fuller M. Neurocognitive testing in a murine model of mucopolysaccharidosis type IIIA. Mol Genet Metab Rep. 2023;36:100985. doi: 10.1016/j.ymgmr.2023.100985 37332488 PMC10276283

[32] Langford-Smith A, Langford-Smith KJ, Jones SA, Wynn RF, Wraith JE, Wilkinson FL, et al. Female mucopolysaccharidosis IIIA mice exhibit hyperactivity and a reduced sense of danger in the open field test. PLoS One. 2011;6(10):e25717. doi: 10.1371/journal.pone.0025717 22028789 PMC3196509

[33] Roca C, Motas S, Marcó S, Ribera A, Sánchez V, Sánchez X, et al. Disease correction by AAV-mediated gene therapy in a new mouse model of mucopolysaccharidosis type IIID. Hum Mol Genet. 2017;26(8):1535–51. doi: 10.1093/hmg/ddx058 28334745

[34] Perlman RL. Mouse models of human disease: an evolutionary perspective. Evol Med Public Health. 2016;2016(1):170–6. doi: 10.1093/emph/eow014 27121451 PMC4875775

[35] Prinz M, Jung S, Priller J. Microglia biology: one century of evolving concepts. Cell. 2019;179(2):292–311. doi: 10.1016/j.cell.2019.08.053 31585077

[36] Borst K, Dumas AA, Prinz M. Microglia: immune and non-immune functions. Immunity. 2021;54(10):2194–208. doi: 10.1016/j.immuni.2021.09.014 34644556

[37] McShane A, Mole SE. Sex bias and omission exists in Batten disease research: systematic review of the use of animal disease models. Biochim Biophys Acta Mol Basis Dis. 2022;1868(11):166489. doi: 10.1016/j.bbadis.2022.166489 35840041

[38] Mauvais-Jarvis F, Bairey Merz N, Barnes PJ, Brinton RD, Carrero J-J, DeMeo DL, et al. Sex and gender: modifiers of health, disease, and medicine. Lancet. 2020;396(10250):565–82. doi: 10.1016/S0140-6736(20)31561-0 32828189 PMC7440877

[39] Schulz A, Ajayi T, Specchio N, de Los Reyes E, Gissen P, Ballon D, et al. Study of intraventricular cerliponase alfa for CLN2 disease. N Engl J Med. 2018;378(20):1898–907. doi: 10.1056/NEJMoa1712649 29688815

[40] Pardridge WM. Drug transport across the blood-brain barrier. J Cereb Blood Flow Metab. 2012;32(11):1959–72. doi: 10.1038/jcbfm.2012.126 22929442 PMC3494002

[41] Abbott NJ, Pizzo ME, Preston JE, Janigro D, Thorne RG. The role of brain barriers in fluid movement in the CNS: is there a “glymphatic” system?. Acta Neuropathol. 2018;135(3):387–407. doi: 10.1007/s00401-018-1812-4 29428972

[42] Kakkis E, McEntee M, Vogler C, Le S, Levy B, Belichenko P, et al. Intrathecal enzyme replacement therapy reduces lysosomal storage in the brain and meninges of the canine model of MPS I. Mol Genet Metab. 2004;83(1–2):163–74. doi: 10.1016/j.ymgme.2004.07.003 15464431

[43] Dickson P, McEntee M, Vogler C, Le S, Levy B, Peinovich M, et al. Intrathecal enzyme replacement therapy: successful treatment of brain disease via the cerebrospinal fluid. Mol Genet Metab. 2007;91(1):61–8. doi: 10.1016/j.ymgme.2006.12.012 17321776 PMC3009387

[44] Calias P, Banks WA, Begley D, Scarpa M, Dickson P. Intrathecal delivery of protein therapeutics to the brain: a critical reassessment. Pharmacol Ther. 2014;144(2):114–22. doi: 10.1016/j.pharmthera.2014.05.009 24854599

[45] Ullman JC, Arguello A, Getz JA, Bhalla A, Mahon CS, Wang J, et al. Brain delivery and activity of a lysosomal enzyme using a blood-brain barrier transport vehicle in mice. Sci Transl Med. 2020;12(545):eaay1163. doi: 10.1126/scitranslmed.aay1163 32461331

[46] Hsu Y-H, Liu R-S, Lin W-L, Yuh Y-S, Lin S-P, Wong T-T. Transcranial pulsed ultrasound facilitates brain uptake of laronidase in enzyme replacement therapy for Mucopolysaccharidosis type I disease. Orphanet J Rare Dis. 2017;12(1):109. doi: 10.1186/s13023-017-0649-6 28595620 PMC5465581

[47] Hui EK-W, Lu JZ, Boado RJ, Pardridge WM. Preclinical studies of a brain penetrating IgG Trojan horse-arylsulfatase fusion protein in the metachromatic leukodystrophy mouse. Mol Genet Metab. 2019;126(2):S77. doi: 10.1016/j.ymgme.2018.12.186

[48] Logan T, Simon MJ, Rana A, Cherf GM, Srivastava A, Davis SS, et al. Rescue of a lysosomal storage disorder caused by Grn loss of function with a brain penetrant progranulin biologic. Cell. 2021;184(18):4651–68.e25. doi: 10.1016/j.cell.2021.08.002 34450028 PMC8489356

[49] Lochhead JJ, Thorne RG. Intranasal delivery of biologics to the central nervous system. Adv Drug Deliv Rev. 2012;64(7):614–28. doi: 10.1016/j.addr.2011.11.002 22119441

[50] Critchley BJ, Gaspar HB, Benedetti S. Targeting the central nervous system in lysosomal storage diseases: Strategies to deliver therapeutics across the blood-brain barrier. Mol Ther. 2023;31(3):657–75. doi: 10.1016/j.ymthe.2022.11.015 36457248 PMC10014236

[51] Arguello A, Mahon CS, Calvert MEK, Chan D, Dugas JC, Pizzo ME, et al. Molecular architecture determines brain delivery of a transferrin receptor-targeted lysosomal enzyme. J Exp Med. 2022;219(3):e20211057. doi: 10.1084/jem.20211057 35226042 PMC8932535

[52] Wu D, Chen Q, Chen X, Han F, Chen Z, Wang Y. The blood–brain barrier: structure, regulation and drug delivery. Sig Transduct Target Ther. 2023;8(1). doi: 10.1038/s41392-023-01481-w PMC10212980 37231000

[53] Archie SR, Al Shoyaib A, Cucullo L. Blood-brain barrier dysfunction in CNS disorders and putative therapeutic targets: an overview. Pharmaceutics. 2021;13(11):1779. doi: 10.3390/pharmaceutics13111779 34834200 PMC8622070

[54] Iwasaki A. Immune regulation of antibody access to neuronal tissues. Trends Mol Med. 2017;23(3):227–45. doi: 10.1016/j.molmed.2017.01.004 28185790 PMC5626569

[55] Ronzitti G, Collaud F, Laforet P, Mingozzi F. Progress and challenges of gene therapy for Pompe disease. Ann Transl Med. 2019;7(13):287–287. doi: 10.21037/atm.2019.04.67 31392199 PMC6642941

[56] Abelleyra Lastoria DA, Keynes S, Hughes D. Current and emerging therapies for lysosomal storage disorders. Drugs. 2025;85(2):171–92. doi: 10.1007/s40265-025-02145-5 39826077

[57] Kohn DB, Chen YY, Spencer MJ. Successes and challenges in clinical gene therapy. Gene Ther. 2023;30(10–11):738–46. doi: 10.1038/s41434-023-00390-5 37935854 PMC10678346

[58] Hwu WL. Gene therapy for ultrarare diseases: a geneticist’s perspective. J Biomed Sci. 2024;31:1–13. doi: 10.1186/S12929-024-01070-1 39138523 PMC11321167

[59] Fitzpatrick Z, Leborgne C, Barbon E, Masat E, Ronzitti G, van Wittenberghe L, et al. Influence of pre-existing anti-capsid neutralizing and binding antibodies on AAV vector transduction. Mol Ther Methods Clin Dev. 2018;9:119–29. doi: 10.1016/j.omtm.2018.02.003 29766022 PMC5948224

[60] Talele SS, Xu K, Pariser AR, Braun MM, Farag-El-Massah S, Phillips MI, et al. Therapies for inborn errors of metabolism: what has the orphan drug act delivered?. Pediatrics. 2010;126(1):101–6. doi: 10.1542/peds.2009-3246 20566615

[61] Platt FM, d’Azzo A, Davidson BL, Neufeld EF, Tifft CJ. Lysosomal storage diseases. Nat Rev Dis Primers. 2018;4(1):27. doi: 10.1038/s41572-018-0025-4 30275469

